# Effects of Garlic on Breast Tumor Cells with a Triple Negative Phenotype: Peculiar Subtype-Dependent Down-Modulation of Akt Signaling

**DOI:** 10.3390/cells13100822

**Published:** 2024-05-11

**Authors:** Federica Brugnoli, Marcello Dell’Aira, Paola Tedeschi, Silvia Grassilli, Marina Pierantoni, Rebecca Foschi, Valeria Bertagnolo

**Affiliations:** 1 Department of Translational Medicine, University of Ferrara, 44121 Ferrara, Italy; bgf@unife.it (F.B.); marcello.dellaira@unife.it (M.D.); marina.pierantoni@unife.it (M.P.); rebecca.foschi@unife.it (R.F.); 2 Department of Chemical, Pharmaceutical and Agricultural Sciences, University of Ferrara, 44121 Ferrara, Italy; tdspla@unife.it; 3 Department of Environmental Sciences and Prevention and LTTA Centre, University of Ferrara, 44121 Ferrara, Italy

**Keywords:** breast cancer, garlic extract, Akt, TNBC subtypes, PDX-derived cells.

## Abstract

Breast cancer includes tumor subgroups with morphological, molecular, and clinical differences. Intrinsic heterogeneity especially characterizes breast tumors with a triple negative phenotype, often leading to the failure of even the most advanced therapeutic strategies. To improve breast cancer treatment, the use of natural agents to integrate conventional therapies is the subject of ever-increasing attention. In this context, garlic (*Allium sativum*) shows anti-cancerous potential, interfering with the proliferation, motility, and malignant progression of both non-invasive and invasive breast tumor cells. As heterogeneity could be at the basis of variable effects, the main objective of our study was to evaluate the anti-tumoral activity of a garlic extract in breast cancer cells with a triple negative phenotype. Established triple negative breast cancer (TNBC) cell lines from patient-derived xenografts (PDXs) were used, revealing subtype-dependent effects on morphology, cell cycle, and invasive potential, correlated with the peculiar down-modulation of Akt signaling, a crucial regulator in solid tumors. Our results first demonstrate that the effects of garlic on TNBC breast cancer are not unique and suggest that only more precise knowledge of the mechanisms activated by this natural compound in each tumor will allow for the inclusion of garlic in personalized therapeutic approaches to breast cancer.

## 1. Introduction

Breast cancer (BC) is the most diagnosed tumor and the leading cause of cancer-related deaths in women worldwide [1]. BC is a highly heterogenous disease, showing differences in morphology, prognosis, and response to therapy, based on both histopathological and molecular characteristics [2,3,4]. BC classification is primarily based on the expression levels of estrogen receptor (ER), progesterone receptor (PR), and human epidermal growth factor receptor 2 (HER2), allowing us to identify groups of breast tumors with distinct characteristics in terms of proliferation, mutated genes, and invasive potential [5]. The most recent classifications identify Luminal-A (ER+/PR+, HER2−), Luminal-B (ER+/PR±, HER2+), HER2-enriched (ER−/PR−, HER2+), and Basal-like (ER−/PR−, HER2−) breast tumors. The latter are characterized by the lack of expression of ER, PR, HER2, and their related genes, and by the up-regulation of genes expressed by basal/myoepithelial cells. They also show a high incidence of *TP53* mutations, losses of *RB1* and *BRCA1*, and elevated activation of the PI3K/AKT pathway. Basal tumors are extremely aggressive, with the worst prognosis among all subtypes, lacking specific target-based therapies currently available for the other subgroups [6,7,8]. They are also the most heterogeneous, and although they are typically referred to as triple negative, up to 10% can be ER+ and/or HER2+, further complicating their response to conventional therapies and, ultimately, their management. Although recent technologies have made it possible to better characterize triple negative breast cancers (TNBCs), which have been divided into various molecular subtypes showing different levels of gene and protein expression and various metabolic and epigenomic profiles [9,10,11,12], these classifications are still not sufficient to effectively treat this group of breast tumors.

The management of TNBCs primarily includes surgical removal, radiotherapy, and/or chemotherapy, often resulting in side effects and in drug resistance, which diminish the therapeutic potential of many interventions. Despite the wide range of novel treatment approaches currently under investigation, including PARP inhibitors, immune- checkpoint inhibitors, and antibody–drug conjugates (ADCs), additional investigations are necessary to identify TNBC patients who could most benefit from them [13,14,15]. Therefore, it is currently of great interest to identify new strategies adjuvating conventional therapies, and there is increasing consideration of the use of natural substances, due to their potent biological activities and mild side effects [16].

Many phytochemical compounds have been extracted from a variety of plant sources, including fruits, vegetables such as cereals, and traditional herbs such as garlic. Garlic (*Allium sativum* L.) has been extensively studied for its antioxidant, hypocholesterolemic, antihypertensive, and antitumoral effects due to its high content of active phytochemicals, such as organosulfur compounds (OSCs) and antioxidants [17,18,19,20]. Garlic OSCs have been identified as functional substances with potential benefits against invasive breast cancer, counteracting the growth, migration, and invasiveness of breast tumor cells [21]. Most studies have been performed on Luminal MCF-7 and on triple negative MDA-MB-231 cell lines, in which individual garlic derivatives have been found to modulate cell cycle- and/or apoptosis-related proteins, as well as invasion/metastasis signaling pathways [22,23,24,25,26]. We recently also revealed the anti-cancer properties of garlic in non-invasive breast tumor cells, and its protective role against their malignant progression induced by low oxygen availability [27,28].

Despite the increasing number of studies on the role of garlic on features of breast tumor cells, they do not consider that tumor heterogeneity could be at the basis of the variable response to this natural compound. To try to deal with this problem, the main objective of this study was to evaluate the anti-tumoral activity of an organosulfur-enriched garlic extract in breast cancer cells with a triple negative phenotype, the most heterogeneous group of breast tumors. Cell lines from patient-derived xenografts (PDXs) originating from different molecular subtypes of TNBC were used, allowing us to reveal a peculiar response of this tumor phenotype to garlic, in terms of both cell response and the involvement of Akt signaling.

## 2. Materials and Methods

All reagents were obtained from Merck KGaA (Darmstadt, Germany) unless otherwise indicated.

### 2.1. Cells and Treatments

The breast cancer-derived MCF7 and MDA-MB-231 cell lines were acquired from the American Type Culture Collection (Rockville, MD, USA) and were cultured in Dulbecco’s modified Eagle’s medium (DMEM, Gibco Laboratories, Grand Island, NY, USA) supplemented with 10% fetal bovine serum (FBS, Gibco Laboratories) and 1% penicillin–streptomycin solution (Gibco Laboratories). Established cell lines from patient-derived xenografts (PDXs) of TNBC breast tumors belonging to different molecular subtypes (HBCX-9, HBCX-17, HBCX-39, and T174) were provided by Xentech (Evry, France) and cultured as previously reported [29]. All cell lines were maintained at 37 °C in a humidified atmosphere and evaluated monthly for mycoplasma and other contaminations.

Homemade hydro-alcoholic garlic extract (GE) was prepared following the procedure described by Petrovic and colleagues [30]. Briefly, 350 g of garlic was crushed in 250 mL 40% ethanol and, after 5 days of extraction in dark conditions at a refrigerated temperature, centrifuged at 2500× *g* for 10 min at 4 °C to obtain the supernatant, as we previously described [27]. The extract was then subjected to lyophilization for 12 h at −56 °C and 0.090 mbar in Christ-ALPHA 1-2 LD plus (Martin Christ Gefriertrocknungsanlagen GmbH, Germany). The obtained powder was stored at −20 °C until use and resuspended in PBS 1X (0.25 g/mL) before treatments.

All cell lines were treated for 72 h with various dilutions (*v*/*v*) of 0.25 g/mL lyophilized garlic extract (GE). To inhibit Akt activity, cells were treated for 24 h with the allosteric Akt inhibitor MK-2206 (#S1078, Selleck Chemicals, Houston, TX, USA), dissolved in DMSO.

In all experimental conditions, cell viability was evaluated with the Trypan Blue Exclusion Test and inverted phase-contrast microscope (Diaphot, Nikon, Melville, NY, USA) analysis.

### 2.2. Apoptosis and Cell Cycle Analysis

Apoptosis was evaluated using the Muse^®^ Annexin V and Dead Cell Kit (Prodotti Gianni Spa, Milan, Itay), according to the instructions of the manufacturer. Briefly, 3 × 10^4^ cells were washed, resuspended in 100 µL of medium containing 8% FBS, and incubated with 100 µL of reagent for 20 min in the dark at room temperature. All the samples were then analyzed with a Muse™ Cell Analyzer.

Cells were analyzed for cycle distribution following a previously reported procedure [27]. Briefly, 5 × 10^5^ cells were fixed with 70% ethanol and incubated in the dark at room temperature for 30 min with 100 μg/mL RNAse and 20 μg/mL PI. The fluorescence of individual nuclei was measured using a FACS Calibur flow cytometer (BD Biosciences, San Josè, CA, USA). The ratios of cells in the G0/G1, S and G2/M phases were calculated by the CellQuest Pro 6.0 software (BD Biosciences), as previously described [27].

### 2.3. Immunochemical Analysis

Total lysates from cells under the different experimental conditions were separated on 7.5% polyacrylamide denaturing gels and blotted onto nitrocellulose membranes (GE Healthcare Life Science, Little Chalfont, United Kingdom), which were reacted with antibodies directed against E-cadherin (sc-7870), Vimentin (sc-373717), and β-Catenin (sc-7963) from Santa Cruz Biotechnology (Santa Cruz, CA, USA); against Akt2 (#3063), p-Akt2 (Ser474, #8599), p-GSK-3β (Ser9, #5558), GSK-3β (#9315), p-p70 S6 Kinase (Thr389, #9205), and p70 S6 Kinase (#9202) from Cell Signaling Technology (Danvers, MA, USA); and against Akt1 (#610860, BD Biosciences), anti-p-Akt1 (Ser473, #05-739), and anti-β-Tubulin (#T4026), following previously reported procedures [27]. The immunocomplexes were detected by using a WESTAR NOVA 2.0 (Cyanagen, Bologna, Italy), and the chemiluminescence-derived bands were captured with an ImageQuantTM LAS 4000 imager (GE Healthcare Life Science) and quantified with Image Quant TL software v7.0 (GE Healthcare Life Science), as previously reported [27].

### 2.4. Immunocytochemical Analysis

Cells grown on glass slides were fixed with freshly prepared 4% paraformaldehyde, reacted with a specific primary antibody against α-Tubulin (#T9026) for 3 h at room temperature in Net Gel solution (150 mM NaCl, 5 mM EDTA, 50 mM Tris-HCl pH 7.4, 0.05% NP40, 0.25% Carrageenan Lambda gelatin, and 0.02% Na azide), and then labeled with an FITC-conjugated secondary antibody (Thermo Fisher Scientific, Rockford, IL, USA) in the dark at room temperature. Subsequently, all samples were incubated with 0.5 µg/mL 4′,6-diamidino-2-phenylindole (DAPI), dried with ethanol, and mounted in glycerol containing 1,4-diazabicyclo [2.2.2] octane (DABCO) to delay fading. Fluorescent samples were analyzed with a Nikon Ci-L microscope (Nikon) and images were captured using a DS-Qi2Mc digital camera equipped with NIS-ELEMENTS software version D (Nikon, Tokyo, Japan). For each condition, digitized images of at least 20 cells from three different areas were examined with 1.53e ImageJ software (http://rsb.info.nih.gov/ij/, accessed on 28 March 2024), and fluorescence values of integrated density (IntDen) were expressed as arbitrary units/cell [31].

### 2.5. Real-Time Cell Migration and Invasion Assays

Cell migration and invasion were evaluated with an xCELLigence Real-Time Cell Analyzer System (RTCA System, Roche Applied Science, Mannheim, Germany), developed to monitor cell events in real time. For each assay, 40,000 cells/well were seeded onto the top chambers of CIM-16 plates (Roche Applied Science). For only the invasiveness experiments, the upper chambers were covered with Matrigel (BD Biosciences) diluted to 1:20, while the bottom chambers were filled with medium containing 5% (migration) or 10% (invasion) FBS (Gibco Laboratories) and the signal detection was automated every 15 min for a total of 24 h, as previously described [32]. Impedance values are shown as the dimensionless parameter Cell Index (CI).

### 2.6. Statistical Analysis

Statistical analysis was performed by using a 2-tailed Student’s t test for unpaired data using the GraphPad Prism 6.0 statistical package (GraphPad Software, San Diego, CA, USA).

The results are expressed as means ± standard deviations of three independent experiments. *p* values < 0.05 are considered statistically significant.

## 3. Results

### 3.1. Effects of Garlic Extract on Growth and Invasive Potential of MCF7 and MDA-MB-231 Breast Tumor Cells

**Figure 1:**
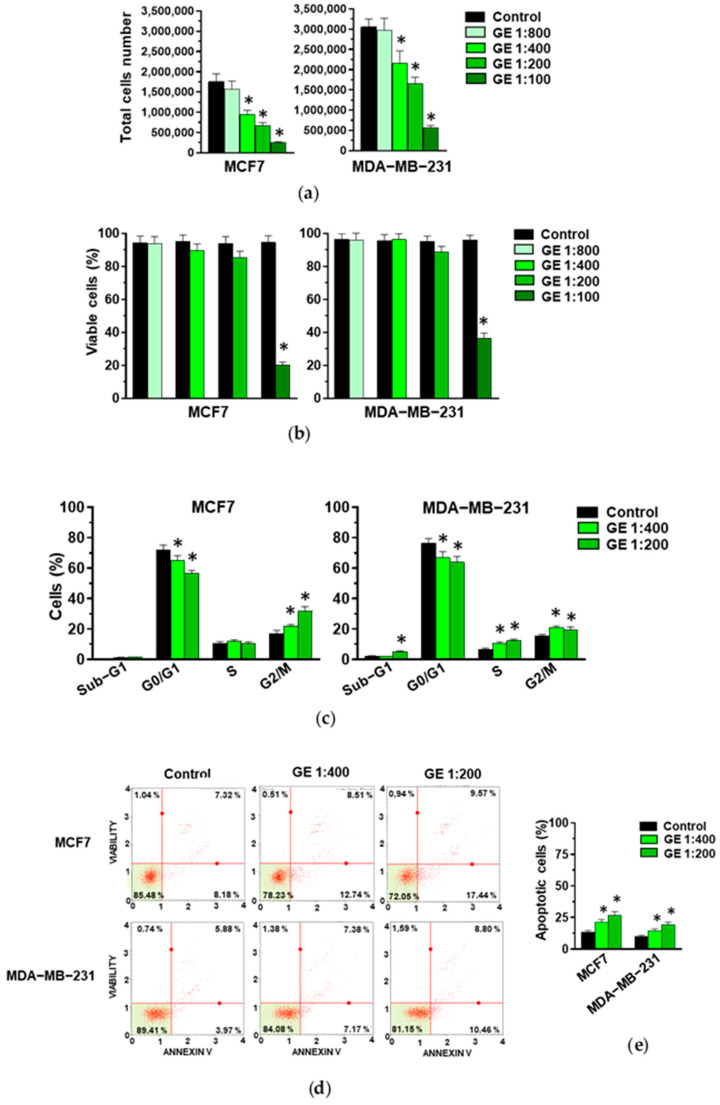
Effect of GE on growth of MCF7 and MDA-MB-231 cells. After 72 hours of culture in control conditions (Control) or in the presence of the reported dilutions of garlic extract (GE), cells were subjected to an evaluation of proliferation (**a**) and the percentage of viable cells (**b**), and to a cytofluorimetric analysis of cell cycle distribution (**c**). (**d**) A representative apoptosis profile obtained after an Annexin V assay on MCF7 and MDA-MB-231 cells under the indicated conditions. (**e**) Percentage of total (early and late) apoptotic cells. Results represent the mean of three experiments ± SD. * *p* < 0.05 compared to control.

Given that most of the studies on the role of garlic derivatives in breast cancer have been conducted on MCF7 and MDA-MB-231, we first assessed the efficacy of our garlic extract (GE) on these cell lines. MCF7 and MDA-MB-231 cells were treated for 72 h with various GE dilutions and subjected to an evaluation of cell growth, to identify the maximum concentration affecting cell growth without inducing excessive toxic effects. We revealed that, apart from the higher dilution, GE administration induced a significant decrease in cell growth in MCF7 cells. On the other hand, a significant decrease in the total cell number was induced by 1:200 and 1:100 GE in MDA-MB-231 cells (Figure 1a). Only 1:100 GE significantly reduced the number of viable MCF7 and MDA-MB-231 cells (Figure 1b), indicative of a high toxic effect of this GE amount. Based on these first results, the 1:800 and 1:100 GE dilutions were excluded from the subsequent experiments with these cell lines.

When the effects of the selected GE amounts (1:400 and 1:200) on cell cycle were evaluated, a significant and dilution-dependent decrease in MCF7 cells in the G0/G1 phase and their accumulation in the G2/M phases of the cell cycle were revealed (Figure 1c), confirming our previously reported data on the effects of our GE on this cell line [27,28]. Significant effects were also detected in the distribution of MDA-MB-231 cells along the cell cycle, with a similar decrease in the G0/G1 and an increase in the S and G2/M phases after the administration of 1:400 and 1:200 GE (Figure 1c).

As garlic derivatives are known to modulate apoptosis in both MCF7 and MDA-MB-231 cells, this process was investigated, revealing significant and dilution-dependent effects of our GE in inducing early and/or late apoptosis in both examined cell lines (Figure 1d,e).

**Figure 2:**
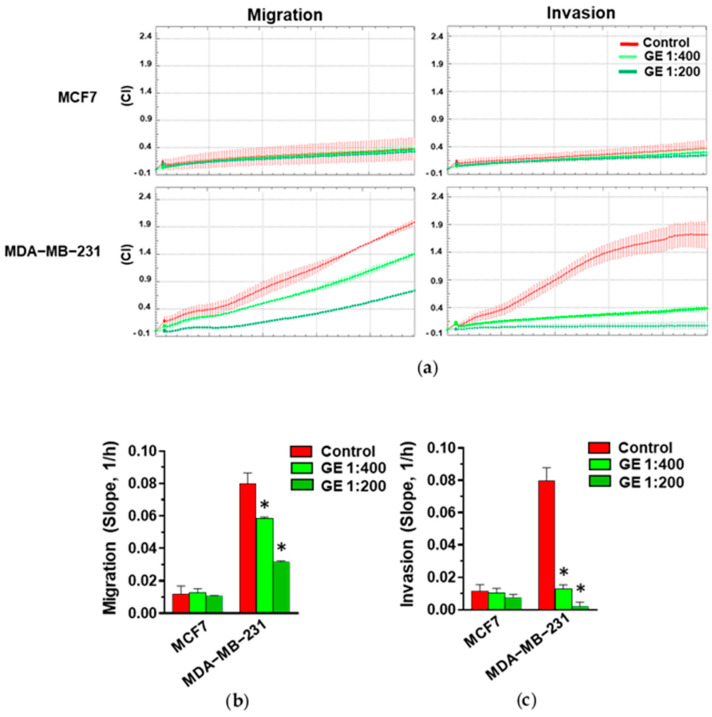
Effect of GE on migration and invasion of breast cancer derived cells. (**a**) Representative xCELLigence-driven dynamic monitoring of migration and invasion of MCF7 and MDA-MB-231 cells after 72 hours in control conditions or in the presence of the indicated GE dilutions. Curves represent mean ± SD of Cell Index (CI). (**b**,**c**) Slope analysis of migration and invasion CI curves, respectively. Data represent the mean of three experiments ± SD. * *p* < 0.05 compared to control.

Based on the known effects of garlic derivatives on breast tumor cell motility, both MCF7 and MDA-MB-231 cells were treated with GE and subjected to real-time migration and invasion assays using the xCELLigence technique. As expected, and according to their phenotype, MCF7 cells exhibited low migratory aptitude and ability to pass through Matrigel with respect to MDA-MB-231 (Figure 2a). Following treatment with GE, no significant effects were detected on the migration and invasion of MCF7 cells, while a significant and dilution-dependent reduction in both migration and invasive potential was revealed in MDA-MB-231 (Figure 2a–c). 

### 3.2. Effects of Garlic extract on Growth and Invasive Potential of PDX-Derived Breast Tumor Cells with a Triple Negative Phenotype

Starting from the notion that TNBC shows intrinsic molecular heterogeneity, our study further aimed to analyze the role of GE on the cell cycle, morphology, and motility of breast cancer cells with a triple negative phenotype belonging to different molecular subtypes. With this purpose, the effects of GE were evaluated on four cell lines established from patient-derived xenografts (PDXs), translational preclinical models that better reflect the heterogeneity and diversity of tumor cell populations [33,34,35,36].

**Figure 3:**
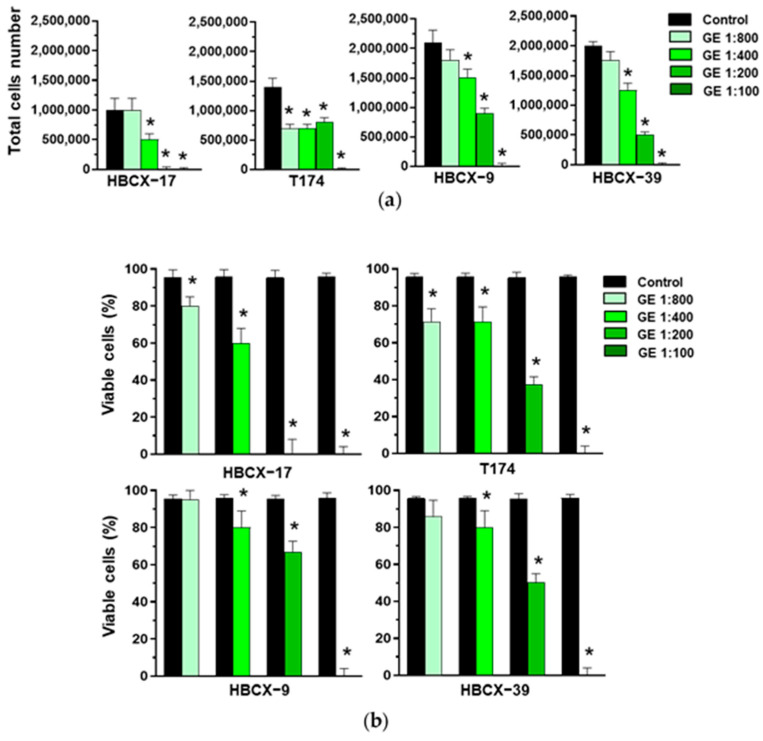
Effect of garlic extracts on growth of HBCX-17, T174, HBCX-9, and HBCX-39 cells. (**a**) Proliferation of the indicated PDX-derived cell lines after 72 hours of culture in control conditions (Control) or in the presence of the reported dilutions of garlic extract (GE). (**b**) Percentage of viable cells under the above-reported conditions. Results represent the mean of three independent experiments ± SD. * *p* < 0.05 compared to untreated cells (Control).

Various GE dilutions were used to treat the HBCX-17, T174, HBCX-9, and HBCX-39 PDX-derived cell lines, revealing different responses, both in terms of cell proliferation and viability. The 1:800 GE dilution down-modulated the total cell number only in T174 cells (Figure 3a), in which it also reduced viability (Figure 3b). On the other hand, 1:100 GE completely abolished the viability of all cell lines, while 1:200 GE induced the death of HBCX-17 cells and reduced the number and viability of the other explored cell lines. As 1:400 GE significantly reduced the number of all cell lines without inducing high toxic effects (Figure 3a,b), this dilution was chosen for the following experiments with PDX-derived cells.

**Figure 4:**
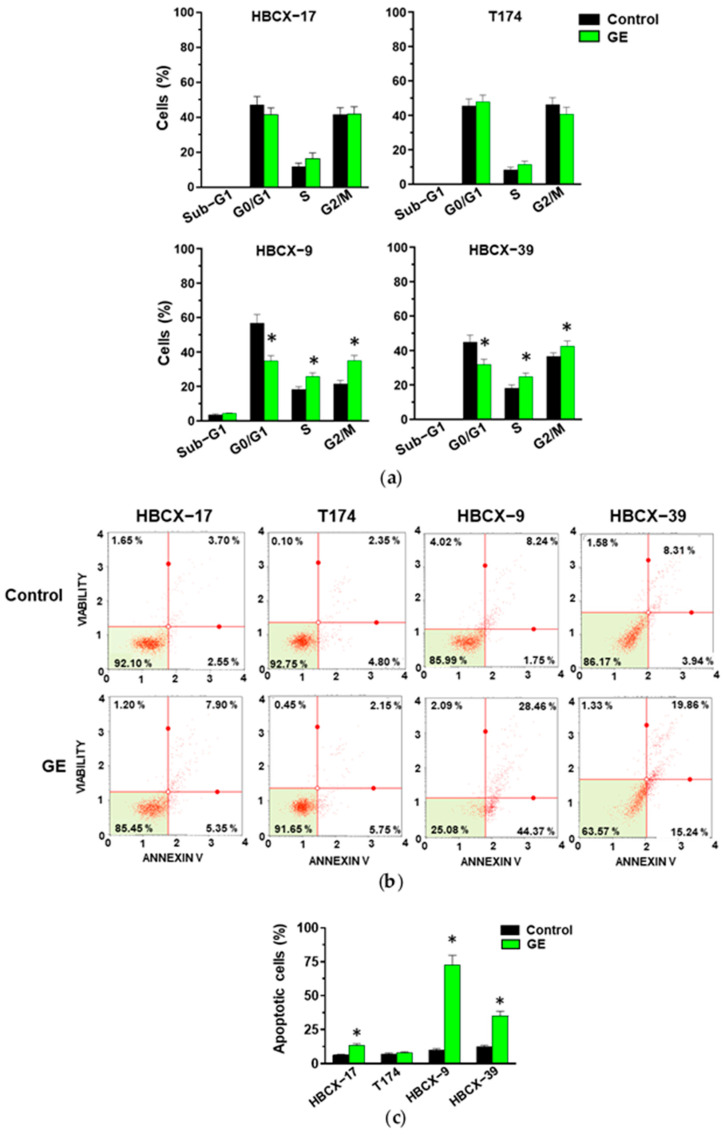
Effect of garlic extracts on cell cycle and apoptosis of PDX-derived cells. (**a**) Cytofluorimetric analysis of cell cycle distribution of HBCX-17, T174, HBCX-9, and HBCX-39 cell lines treated with 1:400 GE for 72 h. (**b**) Muse Cell Analyzer apoptosis profile of the same cells treated as indicated above. (**c**) Percentage of total apoptotic cells. Results represent the mean of three independent experiments ±SD. * *p* < 0.05 compared to control cells.

The analysis of cell cycles showed that GE failed to modify the number of HBCX-17 and T174 cells in the different phases, while in HBCX-9 and HBCX-39 cells, it induced a significant decrease in the number of cells in the G0/G1 phases and an increase in cells in the S and G2/M phases of the cell cycle (Figure 4a).

The analysis with the annexin V failed to reveal significant effects of GE in inducing early (bottom right quadrant) or late (top right quadrant) apoptosis in T174 cells, while the number of apoptotic cells significantly increased in the other examined cell lines, particularly in HBCX-9 (Figure 4b,c).

**Figure 5:**
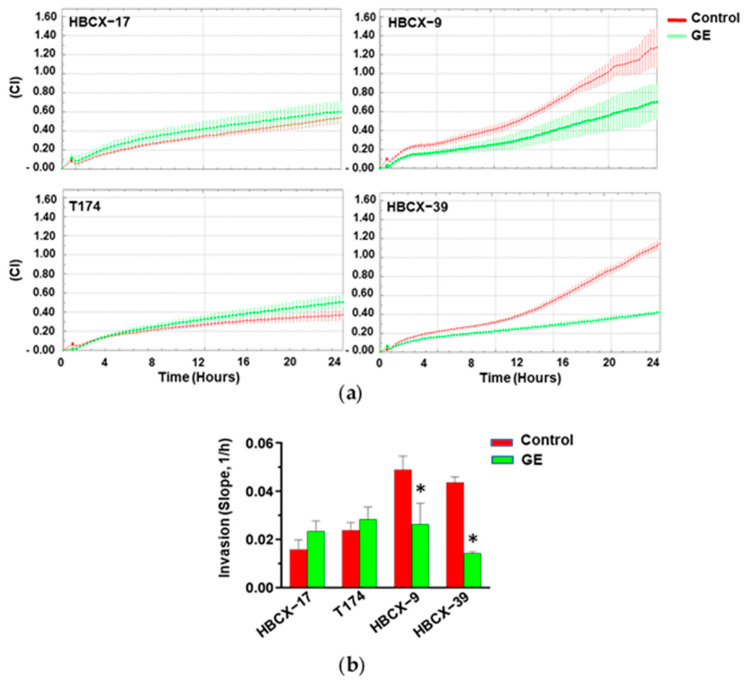
Effect of garlic extract on invasive capabilities of PDX-derived cell lines. (**a**) xCELLigence-driven dynamic monitoring of invasion of HBCX-17, T174, HBCX-9, and HBCX-39 cells treated or not with GE for 72 h. Curves represent mean ± SD of CI. (**b**) Correspondent slope analysis, indicating steepness, inclination, gradient, and changing rate of CI curve over time. Results represent the mean of three independent experiments ± SD. * *p* < 0.05 compared to untreated cells (Control).

To establish whether treatment with our garlic extract may influence the invasive potential of PDX-derived TNBC cells, they were subjected to real-time invasiveness assays, revealing variable ability to pass through Matrigel in control conditions (Figure 5a). The treatment with GE induced an important reduction in the invasiveness of the most invasive HBCX-9 and HBCX-39 cell lines, while no effect of garlic extract was revealed on the invasion capability of the less invasive HBCX-17 and T174 cells (Figure 5a,b).

### 3.3. Effects of Garlic Extract on Epithelial/Mesenchymal Morphology of TNBC Cells

**Figure 6:**
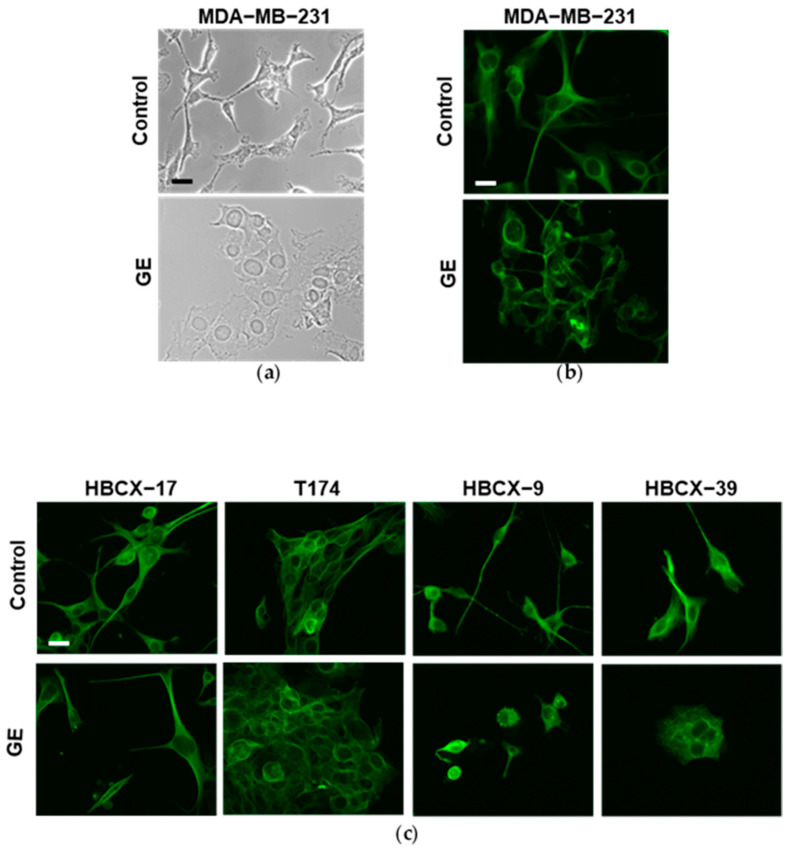
Morphological analysis of MDA-MB-231 and PDX-derived TNBC cell lines after GE treatment. (**a**) Representative phase-contrast image of MDA-MB-231 cells growing on glass dishes for 72 h in control conditions or in the presence of 1:400 diluted GE. (**b**) Representative fluorescence images after immunocytochemical analysis with anti-α-tubulin antibody of MDA-MB-231 cells in the same conditions. (**c**) Representative florescence images of PDX-derived cell lines grown on glass dishes for 72 h in the presence or not of 1:400 diluted GE and then subjected to immunocytochemical analysis of α-tubulin antibody. Scale bar: 50 μm.

Once we established that GE has variable effects on the invasive potential of TNBC cells, we explored the possibility that this may be due to a different effect on the cell phenotype. The morphology of all examined TNBC cells cultured with GE for 72 h was then evaluated, together with the expression of some markers of the epithelial-to-mesenchymal transition (EMT). As shown in Figure 6a, under control conditions, the MDA-MB-231 cells exhibited the well-known fibroblast-like morphology, with cytoplasmic extensions consistent with their high motility. In this cell line, treatment with GE induced a substantial change in morphology and the cells acquired a more polygonal appearance, with a reduction in cytoplasmic extensions and the partial loss of contact inhibition. To better highlight the effects of GE on morphology, we performed immunocytochemical evaluation of α-Tubulin, a cytoskeletal protein localized in microtubules, and fundamental in maintaining cell shape, in intracellular transport and in cell division [36]. As shown in Figure 6b, α-Tubulin perfectly draws the cell morphology, confirming that GE reduced the cytoplasmic extensions of MDA-MB-231, indicative of its effects on the motility of these cells. Immunocytochemical analysis of α-Tubulin was also performed on PDX-derived cells, showing variously elongated shapes in control conditions, with T174 cells displaying a more polygonal profile and close growth (Figure 6c). GE treatment significantly reduced elongations of HBCX-9 and HBCX-39 cells, and induced the contact inhibition of HBCX-39, growing in large monolayer clusters, while no evident effects were observed on the morphology of HBCX-17 and T174 cells (Figure 6c). 

**Figure 7:**
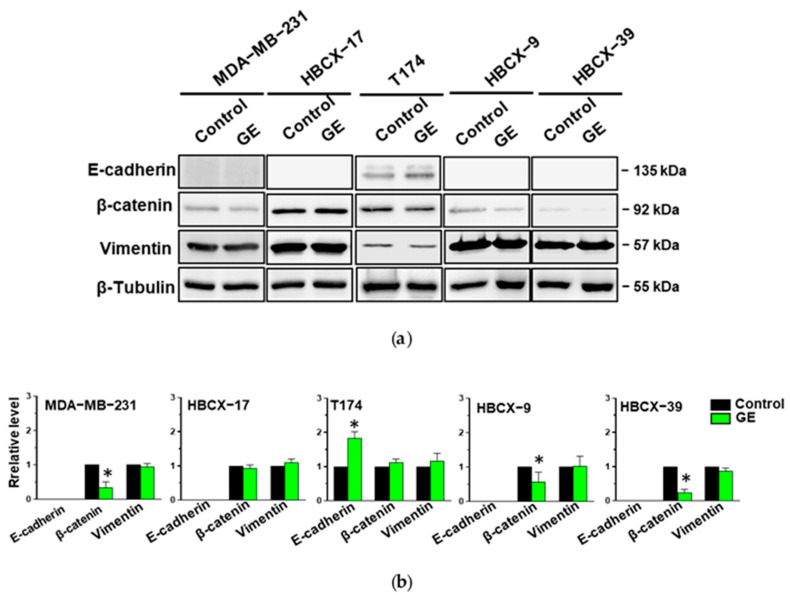
Effect of garlic extract on EMT markers in MDA-MB-231 and PDX-derived TNBC cell lines. (**a**) Representative Western blot analysis with the indicated antibodies of total lysates from the reported cell lines treated with GE for 72 h. β-Tubulin was used as internal control for equivalence of loaded proteins. (**b**) Histograms showing protein levels, normalized using β-Tubulin, as deduced from band densitometry obtained through chemiluminescence signal. Asterisks indicate statistically significant differences with respect to corresponding controls taken as 1. Results represent the mean of three independent experiments ± SD. * *p* < 0.05 compared to control cells.

Considering that garlic extract can partially reduce the fibroblastoid shape of MDA-MB-231, HBCX-9, and HBCX-39 cells, some of the molecules strongly associated with the epithelial-to-mesenchymal transition were evaluated. The epithelial marker E-cadherin is expressed in control conditions only in T174 cells, showing a less elongated morphology. GE treatment induced a significant increase in E-cadherin in T174, but was unable to induce the expression of this epithelial marker in the other examined TNBC cells. All cell lines express the mesenchymal marker Vimentin, in accordance with their basal-like phenotype, which was not significantly down-modulated by GE treatment. All examined TNBC cell lines express the mesenchymal marker β-Catenin, which was significantly down-modulated by GE, except for HBCX-17 and T174, in which garlic extract also failed to modify the morphology (Figure 7).

### 3.4. Effects of GE on Akt in TNBC Cells

As we have demonstrated above that our garlic extract regulates the morphology, proliferation, apoptosis, and migration/invasiveness of MDA-MB-231 and of some PDX-derived TNBC cell lines, we investigated the intracellular signaling molecules potentially underlying these phenomena. Therefore, the effects of GE were evaluated on Akt, a family of proteins highly dysregulated in breast tumors, and implicated in the control of the cell cycle and invasive potential, as well as in the EMT process [37].

**Figure 8:**
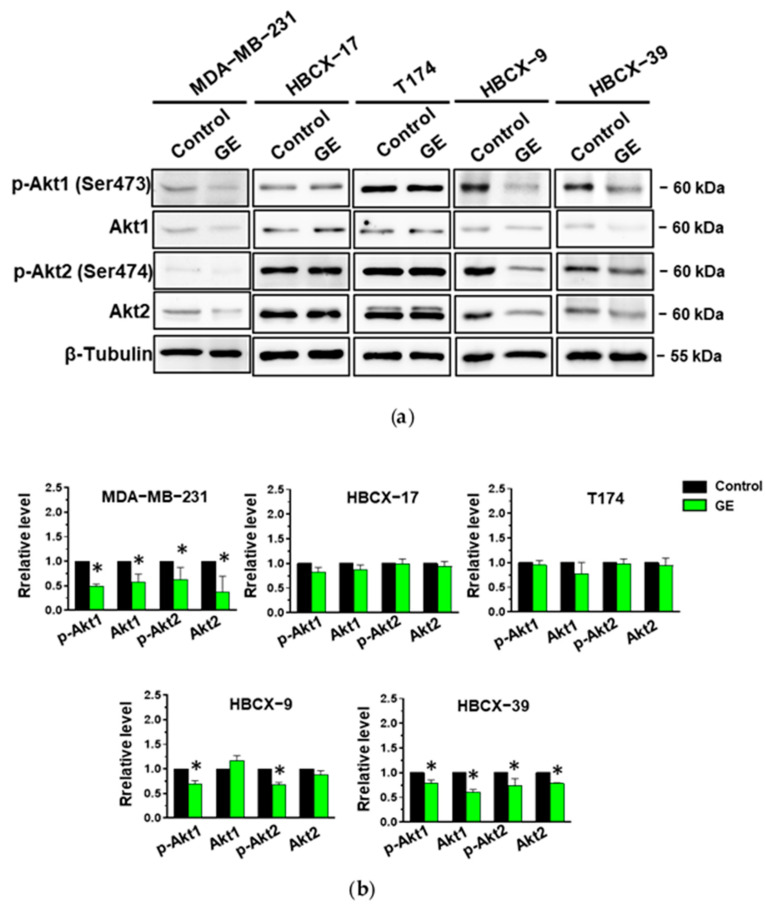
Effect of garlic extract on Akt status in MDA-MB-231 and in PDX-derived cell lines. (**a**) Representative immunoblot analysis with the indicated antibodies of total lysates from MDA-MB-231 and PDX-derived cells after 72 hours of culture in control conditions (Control) or in the presence of 1:400 diluted GE. (**b**) Histograms, as deduced from the densitometry of Western blot bands, reporting the levels of the indicated proteins normalized to β-Tubulin. Results represent the mean of three independent experiments ± SD. * *p* < 0.05 compared to control cells taken as 1.

The TNBC cell lines under investigation were then treated with garlic extract and first subjected to immunochemical analysis of the expression and activation status of Akt1 and Akt2, the most studied isozymes in breast tumors. As shown in Figure 8a, the use of specific antibodies revealed that all cell lines grown in control conditions express Akt1 phosphorylated on Ser473, and Akt2 phosphorylated on Ser474. Our garlic extract induced a significant decrease in both the expression and phosphorylation of the two Akt isoforms in the MDA-MB-231 and HBCX-39 cell lines (Figure 8a,b). A significant reduction in only the activation status of both isoforms was induced by GE in HBCX-9, while GE administration failed to affect the expression and phosphorylation of the examined Akt isoforms in HBCX-17 and T174 cells (Figure 8a,b), according to its lack of effect on the cell cycle, apoptosis, and invasiveness of these cell lines.

**Figure 9:**
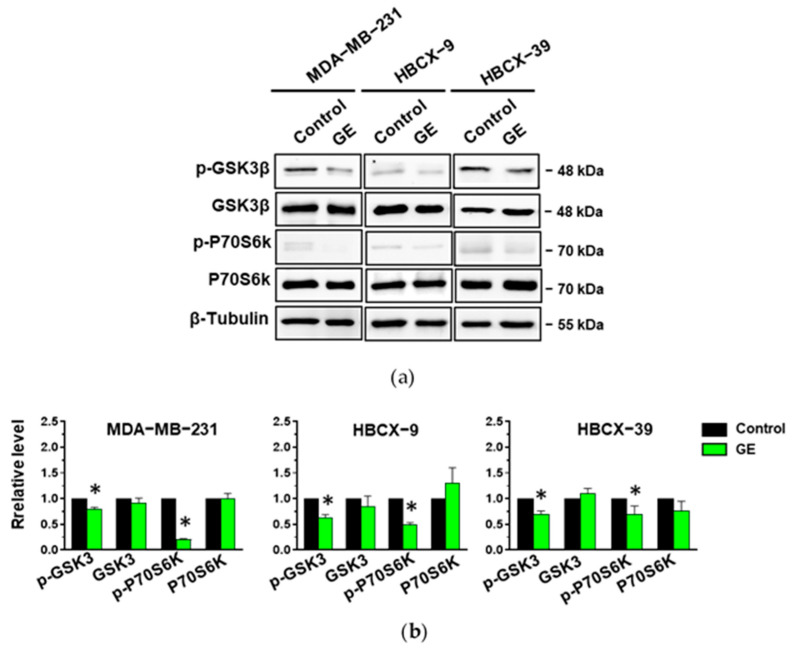
Effect of garlic extract on Akt targets in MDA-MB-231 and in PDX-derived cell lines. (**a**), representative immunoblot analysis with the indicated antibodies of lysates from MDA-MB-231 and PDX-derived cells after 72 hours of culture in control conditions (Control) or in the presence of garlic extract (GE). (**b**) Levels of proteins normalized to β-Tubulin, as deduced from the densitometry of Western blot bands. Results represent the mean of three independent experiments ± SD. * *p* < 0.05 compared to untreated cells (Control) taken as 1.

To verify if the down-modulation of Akt1 and Akt2 phosphorylation status in MDA-MB-231, HBCX-9, and HBCX-39 cells corresponds to an effective reduction in their activity, we evaluated the activation and the expression of downstream targets involved in the regulation of the cell cycle, apoptosis, and motility in TNBC cells [38]. As reported in Figure 9, all three cell lines show Ser9-phosphorylated GSK-3β, an enzyme involved in the regulation of EMT-dependent migratory capacities [39] and in the control of β-catenin degradation [40], and Thr389 phosphorylation of p70S6K, involved in cell growth and motility [41]. We revealed that GE administration induced a significant reduction in GSK-3β and p70 S6K activation without affecting their amount (Figure 9a,b).

**Figure 10:**
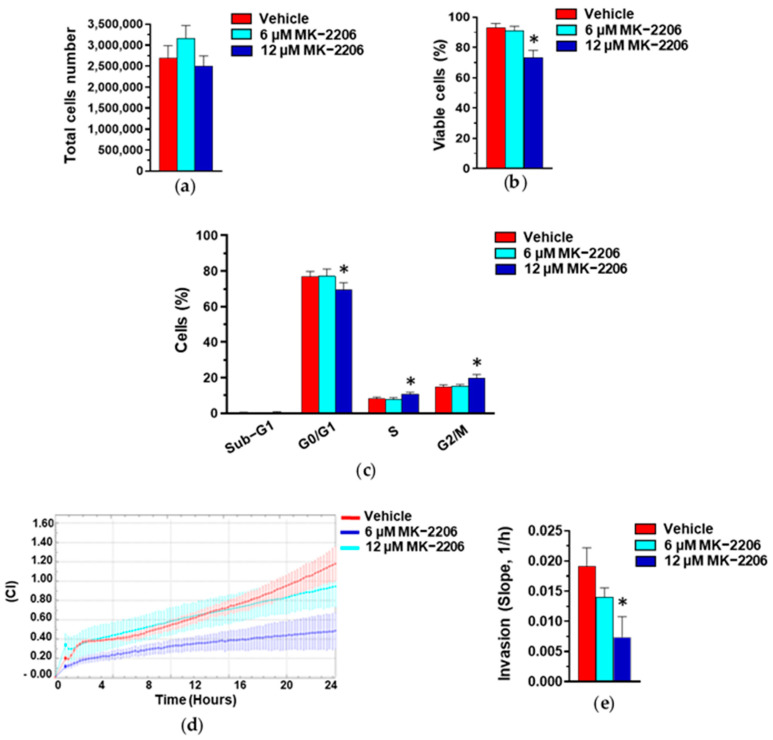
Effect of Akt inhibition on MDA-MB-231 cells. Proliferation (**a**), percentage of viable cells (**b**), cytofluorimetric analysis of cell cycle distribution (**c**), and invasive potential (**d**,**e**) of MDA-MB-231 cells after 24 hours of culture in control conditions (Vehicle) or in the presence of the reported concentrations of MK-2206. Results represent the mean of three independent experiments ± SD. * *p* < 0.05 compared to control cells (Vehicle).

To confirm that the Akt activation status is linked to growth and invasive potential in our cell model, we used the highly selective Akt inhibitor MK-2206, inducing a substantial decrease in Akt phosphorylation in MDA-MB-231 cells [42,43]. As reported in Figure 10, a MK-2206 concentration inducing low toxicity significantly increased the number of cells in the S and G2/M phases of the cell cycle and induced a substantial decrease in the invasive potential of MDA-MB-231 cells, mimicking the effects of our GE in this cell line.

## 4. Discussion

Among the natural compounds used in the treatment of various pathologies, garlic and its derivatives have proven useful in the prevention of breast cancer, as well as in inducing apoptosis and/or in reducing the invasive potential of human breast tumor cells in vitro and of animal tumor models in vivo [22,24,25,26]. This suggests that the possibility of using garlic derivatives as adjuvants in the treatment of breast tumors that do not present satisfactory therapeutic approaches is not far away. However, despite increasing encouraging results, no literature data provide information regarding possible different responses of tumors with the same histopathological phenotype.

Here, we used a hydroalcoholic garlic extract containing a mixture of the most active organosulfur compounds, such as diallyl sulfide (DAS) and diallyl disulfide (DADS) [28], to investigate the effects of this natural compound on different molecular subtypes of triple negative tumors. We first demonstrated that our GE blocks cell cycle at the G2/M phases, induces apoptosis, and reduces the invasiveness of MDA-MB-231 cells, in line with the effects described for individual garlic derivatives in this cell line [22,25]. We then investigated the effects of GE in four cell lines established from patient-derived xenografts (PDXs), preclinical models that maintain the characteristics of primary tumors in terms of the intraphenotypic heterogeneity of tumor cell populations [33,34,35]. We used the HBCX-17, T174, HBCX-9, and HBCX-39 cell lines, which, despite their common basal-like phenotype, belong to different TNBC molecular subtypes, according to the last Lehmann classification, also considering the response to neoadjuvant chemotherapy and disease progression [9,44]. We revealed that in HBCX-17 and T174 cells, belonging to the mesenchymal subtype (M), GE has a strong toxic effect, without significantly affecting the cell cycle and invasive potential. Regarding variance, in the mesenchymal (M) HBCX-9, and in HBCX-39, classified as basal-like 1 (BL1) by Coussy et al. [44], a modest toxic concentration of GE blocked the cell cycle, induced apoptosis, and reduced invasion capability, as in mesenchymal MDA-MB-231 cells [45].

The reduced invasive potential of MDA-MB-231, HBCX-9, and HBCX-39 cells is in line with the GE-induced modification of their elongated shape in favor of a more polygonal profile. This event is accompanied by a decrease in β-catenin, a potent oncoprotein that promotes malignant transformation by increasing migratory capability and invasiveness [46]. These data agree with the reported down-modulation of the β-catenin/Wnt signaling pathway and metastasis induced by DADS in TNBC cells [47]. Despite its elongated morphology, T174 is the only examined PDX-derived cell line expressing E-cadherin, which increased after GE administration, according to the reported effects of GE derivatives on this epithelial marker in breast tumor cells [18,23]. In this cell line, in which β-catenin was not modified, GE was unable to reduce migration and/or invasiveness, supporting previous data suggesting that the antitumor effect of some garlic derivatives on TNBC cells is mediated by the β-catenin pathway [26,47].

Concerning the mechanism activated by garlic derivatives in cancers, the crucial Akt signaling transducer in tumors [48] was explored in T24 bladder cancer cells, in which diallyl trisulfide (DATS) induced G2/M phase cell cycle arrest and apoptosis through the inhibition of PI3K/Akt [49]. Similar effects are induced by DATS in human gastric cancer BGC-823 cells in vitro, and in BGC-823 xenografts in vivo, by attenuating the Nrf2/Akt pathway [50]. More recently, it has been reported that DATS significantly inhibits proliferation, migration, and EMT, promotes cell apoptosis, and induces autophagy in osteosarcoma cells trough the inactivation of the EGFR/PI3K/AKT/mTOR pathway [51]. DADS plays anti-apoptotic or anti-metastatic effects through inhibition of the PI3K/Akt signaling pathways in DU145 prostate cancer cells [52] and in esophageal–gastric junction OE19 cells [53].

Unlike other tumors, few data have correlated Akt to cell growth and apoptosis in breast cancer. Malki et al. [54] first reported that DATS induced the apoptotic cell death of MCF-7 and MCF-12a cells by downregulating the expression levels of Akt and Bcl-2. Recently, allicin was reported to induce apoptosis and reduce Nrf2, HO-1, and p-Akt levels in doxorubicin-resistant MCF-7 and MDA-MB-231 cells, supporting the potential of this garlic compound in improving the effects of this chemotherapeutic in breast cancer [55]. As our garlic extract-modified cell events variously correlated with Akt signaling, we explored the Akt status in TNBC cells, focusing on Akt1 and Akt2, which, in breast cancer, mainly regulate cell growth and invasion/metastasis, respectively [56,57]. Significant decreases in the serine phosphorylation of both Akt1 and Akt2 were revealed after GE administration in MDA-MB-231, HBCX-9, and HBCX-39 cells, in which garlic extract induced effects on the morphology, invasiveness, and expression levels of β-catenin. This clearly suggests that Akt signaling is at the basis of the effects of GE on these TNBC cells. As in MDA-MB-231 and HBCX-39, a decrease in proteins levels was also revealed, different mechanisms could be activated by GE to regulate Akt1 and Akt2 status in TNBC cells. The lack of observed Akt modifications in the HBCX-17 and T174 cells, in which GE did not modify cell cycle, invasive potential, and β-catenin, on one hand, supports the role of Akt in modulating these cellular events activated by garlic extract in MDA-MB-231, HBCX-9, and HBCX-39 cells, and on the other, suggest that the mechanisms leading to the activation of Akt signaling are altered in these two cell lines.

The effects of our GE on Akt activity were confirmed by an evaluation of some of the know targets of this protein family [48]. In the MDA-MB-231, HBCX-9, and HBCX-39 cell lines, in which the activation of Akt was reduced, GE was also responsible for down-modulating the Thr389 phosphorylation of the ribosomal p70S6K, one of the mTORC1 targets known to regulate cell growth, and often amplified in triple negative breast tumors, in which it was correlated with proliferation, migration, and metastasis [58]. In the same cells, we further detected the down-modulation of the Ser9 phosphorylation of GSK-3β, a kinase constitutively active in the cells, which induces cyclin D1 ubiquitination and degradation [40]. In tumors, GSK-3β is also responsible for inhibiting *cyclin D1* gene expression by disrupting the β-catenin/TCF complex, known to bind to the *cyclin D1* promoter [40]. Akt phosphorylation at S9 inactivates GSK-3β, promoting cyclin D1 expression and stability and inducing cell cycle progression. Active GSK-3β also results in the phosphorylation, followed by the degradation, of β-catenin, while GSK-3β inactivation by Akt increases the stability of both β-catenin and Snail, which play a crucial role in tumor progression [40]. Also, p70S6K can phosphorylate and inactivate GSK-3β, which, in turn, is responsible for p70S6K activation [40], and GSK-3β is considered a key regulatory molecule in the sensitivity of breast cancer cells to chemo-, hormonal, and targeted therapy [59]. Overall, our data suggest that, in TNBC cells with certain characteristics, GE down-modulates cell growth and invasive potential through the downregulation of Akt, leading to an increase in GSK-3β activity, which, in turn, is responsible for decreased β-catenin levels and cell cycle blockage, sustaining the role of GSK-3β as a node for crosstalk between the Wnt/β-catenin and PI3K/PTEN signaling pathways.

The various effects of our GE on Akt in the examined PDX-derived cell lines are apparently not related to their molecular subtypes, but could be explained by their different gene alterations, particularly by key mutations identified in their genome. In fact, as reported following the characterization of the examined cell lines [https://www.xentech.eu/models/pdx-derived/, accessed on 28 March 2024], they are all characterized by mutations in the gene for P53, while only HBCX-17 and T174 cells have mutations in the genes for Akt1 and PI3K, respectively, which could compromise the involvement of PI3K/Akt signaling. In addition, deletion in the gene for CDKN2A (Cyclin-Dependent Kinase Inhibitor 2A), capable of inducing cell cycle arrest in the G2 phase and apoptosis by preventing the activation of cyclin B1/CDC2 complexes, and acting as a tumor suppressor in breast cancer [60], is common in HBCX-17 and T174 cells. Noteworthy is the lack of the reported mutations in the classification of HBCX-17, HBCX-9, and HBCX-39 cells [44], indicating that ever-new information improves our knowledge of these cellular models. As mutation-based signatures have been recently proposed as discriminators for therapeutic targeting in cancer [61,62], our findings suggest that garlic derivatives could be useful in developing new mutation-related treatments in TNBC.

## 5. Conclusions

In the last ten years, various stratification systems reflecting intrinsic differences between TNBC tumors have improved clinical outcomes, but the treatment of this aggressive breast tumor still needs therapeutic alternatives. Our data, obtained with TNBC cell lines established from PDXs, useful pre-clinical models to study drug efficacy, highlighted how even natural compounds, such as garlic derivatives, widely used in both in vitro and in vivo studies, may have different effects on tumors with the same histopathological phenotype but differing in their molecular subtypes and genomic alterations. This also suggests that studies on the efficacy of natural compounds should use adequate models and consider the possible heterogeneity of different tumors. This would allow researchers to obtain more precise information about their efficiency, which could increase the possibility of their targeted use in adjuvant therapies for breast cancer and other neoplasms.

## Data Availability

All data are contained within the article.
